# Association of outcome with left ventricular volumes and ejection fraction measured with two- and three-dimensional echocardiography in patients referred for routine, clinically indicated studies

**DOI:** 10.3389/fcvm.2022.1065131

**Published:** 2022-12-22

**Authors:** Denisa Muraru, Sorina Mihaila Baldea, Davide Genovese, Michele Tomaselli, Francesca Heilbron, Mara Gavazzoni, Noela Radu, Caravita Sergio, Claudia Baratto, Francesco Perelli, Emanuele Curti, Gianfranco Parati, Luigi P. Badano

**Affiliations:** ^1^Department of Medicine and Surgery, University of Milano-Bicocca, Milan, Italy; ^2^Department of Cardiology, Istituto Auxologico Italiano, IRCCS, Milan, Italy; ^3^Department of Cardiology, University of Medicine and Pharmacy Carol Davila, Bucharest, Romania; ^4^Cardiology Unit, Cardio-Neuro-Vascular Department, Ca’ Foncello Hospital, Treviso, Italy; ^5^Department of Management, Information and Production Engineering, University of Bergamo, Dalmine, Italy

**Keywords:** three-dimensional echocardiography, two-dimensional echocardiography, left ventricular volumes, left ventricular ejection fraction, outcome

## Abstract

**Objectives:**

We sought to analyze if left ventricular (LV) volumes and ejection fraction (EF) measured by three-dimensional echocardiography (3DE) have incremental prognostic value over measurements obtained from two-dimensional echocardiography (2DE) in patients referred to a high-volume echocardiography laboratory for routine, clinically-indicated studies.

**Methods:**

We measured LV volumes and EF using both 2DE and 3DE in 725 consecutive patients (67% men; 59 ± 18 years) with various clinical indications referred for a routine clinical study.

**Results:**

LV volumes were significantly larger, and EF was lower when measured by 3DE than 2DE. During follow-up (3.6 ± 1.2 years), 111 (15.3%) all-cause deaths and 248 (34.2%) cardiac hospitalizations occurred. Larger LV volumes and lower EF were associated with worse outcome independent of age, creatinine, hemoglobin, atrial fibrillation, and ischemic heart diseases). In stepwise Cox regression analyses, the associations of both death and cardiac hospitalization with clinical data (CD: age, creatinine, hemoglobin, atrial fibrillation, and ischemic heart disease) whose Harrel’s C-index (HC) was 0.775, were augmented more by the LV volumes and EF obtained by 3DE than by 2DE parameters. The association of CD with death was not affected by LV end-diastolic volume (EDV) either measured by 2DE or 3DE. Conversely, it was incremented by 3DE LVEF (HC = 0.84, *p* < 0.001) more than 2DE LVEF (HC = 0.814, *p* < 0.001). The association of CD with the composite endpoint (HC = 0.64, *p* = 0.002) was augmented more by 3DE LV EDV (HC = 0.786, *p* < 0.001), end-systolic volume (HC = 0.801, *p* < 0.001), and EF (HC = 0.84, *p* < 0.001) than by the correspondent 2DE parameters (HC = 0.786, HC = 0.796, and 0.84, all *p* < 0.001) In addition, partition values for mild, moderate and severe reduction of the LVEF measured by 3DE showed a higher discriminative power than those measured by 2DE for cardiac death (Log-Rank: χ^2^ = 98.3 vs. χ^2^ = 77.1; *p* < 0.001). Finally, LV dilation defined according to the 3DE threshold values showed higher discriminatory power and prognostic value for death than when using 2DE reference values (3DE LVEDV: χ^2^ = 15.9, *p* < 0.001 vs. χ^2^ = 10.8, *p* = 0.001; 3DE LVESV: χ^2^ = 24.4, *p* < 0.001 vs. χ^2^ = 17.4, *p* = 0.001).

**Conclusion:**

In patients who underwent routine, clinically-indicated echocardiography, 3DE LVEF and ESV showed stronger association with outcome than the corresponding 2DE parameters.

## Introduction

Left ventricular (LV) volumes and ejection fraction (EF) are key parameters to establish a diagnosis and stratify the prognosis in patients with various cardiac conditions (1–5). Moreover, important treatment decisions and evaluation of therapeutic effects are based on these parameters (6–9). Although several imaging techniques can be used to measure LV geometry and function, two-dimensional echocardiography (2DE) represents by far the most frequent imaging modality to obtain LV volumes and EF for both clinical and research purposes. However, 2DE calculations are hampered by view acquisition errors (i.e., view foreshortening), taking into account the function of a limited amount of LV myocardium, and reliance on fixed geometrical assumptions about the geometry of the LV, all of them affecting both the accuracy and the reproducibility of volume calculations (10–13).

The introduction of three-dimensional echocardiography (3DE) in the clinical routine represented a change in paradigm in clinical echocardiography. 3DE overcomes the geometric assumptions about LV geometry, considers the contribution of the whole myocardial shell to LV EF, and enables an accurate and reproducible measurement of LV volumes and EF to be used to manage patient (14). Several studies have shown that 3DE measurements of LV volumes are significantly more accurate than 2DE calculations when compared with cardiac magnetic resonance as the reference imaging modality (10, 15). Accordingly, the American Society of Echocardiography and the European Association of Cardiovascular Imaging have published guidelines about the acquisition and postprocessing of 3DE datasets of the LV, and the recently published guidelines for the cardiac chamber quantification with echocardiography recommends, whenever feasible, the 3DE measurement of LV volumes and EF (16, 17).

Since then, several studies have reported the additive prognostic power of LV volumes and EF measured by 3DE over those calculated by 2DE (18–20). However, despite all these pieces of evidence, the use of 3DE for the assessment of LV volumes and LVEF is not widespread in the clinical arena, yet. Indeed, the added value of 3DE over 2DE parameters describing the LV geometry and function on the prediction of patients’ outcome remains to be clarified in the clinical routine of the echocardiography laboratory.

Accordingly, the aim of our study was to test the hypothesis that LV volumes and EF measured with 3DE has an incremental value over 2DE in predicting outcome in routine patients referred for clinically indicated echocardiography studies.

## Materials and methods

### Study design

We performed a single center, prospective analysis of retrospectively acquired echocardiographic studies obtained from both in- and out-patients performed from October 2018 to December 2021 at our laboratory. Exclusion criteria were age less than 18 years, lack of 3DE acquisitions or incomplete 2DE data for LV volume quantitation, poor quality of the either 2DE or 3DE acquisitions (defined as the impossibility to visualize the endocardium of two or more adjacent LV myocardial segments without the use of contrast agents), echocardiographic studies performed for non-clinical indications (e.g., driving license, sports activity screening, etc.), and lack of follow-up data.

Clinical information at the time of the echocardiographic study included patients risk factors such as hypertension (either blood pressure >140/90 mm Hg or active antihypertensive treatment), hypercholesterolemia (LDL cholesterol >130 mg/dl or active statin treatment), diabetes (fasting glucose > 126 mg/dl), serum levels of creatinine, hemoglobin, atrial fibrillation, and history of ischemic heart disease (previous myocardial infarction or documented coronary artery disease). Data were obtained from the clinical records of our hospital (Table 1). This retrospective analysis of prospectively acquired data was approved by the Ethics Committee of the Istituto Auxologico Italiano, IRCCS (record #2021_05_18_13, approved on May 18, 2021). The need for patient written informed consent was waived due to the retrospective nature of the acquisitions.

**TABLE 1:** Clinical and echocardiographic data of the whole study population and their comparison between patients who died and those who survived.

	Study cohort (*n* = 725)	All-cause deaths (*n* = 111)	Survivors (*n* = 614)	*P*-value
Age, years	59 ± 18	69 ± 16	57 ± 18	**0.025**
Male, *n*(%)	487 (67)	75 (68)	412 (67)	0.852
Heart rate, bpm	70 ± 15	74 ± 19	69 ± 15	**0.003**
Systolic blood pressure, mm Hg	125 ± 20	120 ± 20	125 ± 19	0.813
Diastolic blood pressure, mm Hg	74 ± 11	71 ± 2	75 ± 11	0.906
Body mass index > 25 Kg/m^2^, *n* (%)	365 (50)	330 (51)	35 (42)	0.113
Hypertension, *n* (%)	401 (55)	356 (56)	45 (54)	0.831
Smoking, *n* (%)	259 (36)	234 (36)	25 (30)	0.258
Diabetes, *n* (%)	113 (16)	92 (14)	21 (25)	**0.010**
Dyslipidemia, *n* (%)	306 (42)	273 (43)	33 (40)	0.631
Chronic kidney disease, *n* (%)	363 (50)	296 (46)	67 (81)	<**0.001**
Anemia, *n* (%)	211 (29)	159 (25)	52 (63)	<**0.001**
Atrial fibrillation, *n* (%)	133 (18)	102 (16)	31 (37)	<**0.001**
Ischemic heart disease, *n* (%)	178 (25)	142 (22)	36 (43)	<**0.001**
2DLeft ventricular end-diastolic volume, ml/m^2^	73 ± 28	85 ± 42	71 ± 24	<**0.001**
2DLeft ventricular end-systolic volume, ml/m^2^	36 ± 24	50 ± 37	33 ± 20	<**0.001**
2DLeft ventricular stroke volume, ml/m^2^	37 ± 10	35 ± 11	37 ± 10	0.499
2D Left ventricular ejection fraction,%	54 ± 12	46 ± 14	55 ± 11	<**0.001**
3DLeft ventricular end-diastolic volume, ml/m^2^	81 ± 30	94 ± 43	78 ± 26	<**0.001**
3DLeft ventricular end-systolic volume, ml/m^2^	42 ± 28	59 ± 42	38 ± 23	<**0.001**
3DLeft ventricular stroke volume, ml/m^2^	39 ± 11	35 ± 10	40 ± 71	0.359
3D Left ventricular ejection fraction,%	52 ± 13	43 ± 16	54 ± 12	<**0.001**

Bold values represent the statistically significant.

### Echocardiography

All 2DE and 3DE acquisitions were obtained during the same echocardiographic study using a commercially available echocardiography system (Vivid E95, GE Healthcare, Horten, NO) equipped with both standard 2DE (M5S) and 3DE (4Vc) probes. All echocardiography studies were stored in a digital archive to be exported and analyzed offline using a commercially available software (Echopac BT204, GE Healthcare, Horten, NO).

**FIGURE 1:**
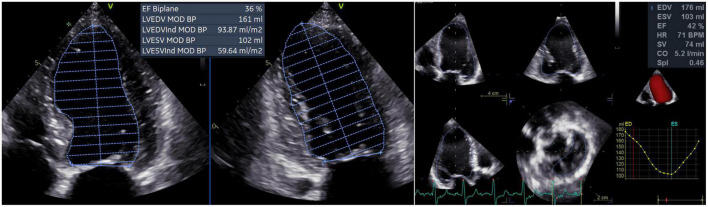
Left ventricular volumes and ejection fraction calculated by two-dimensional echocardiography **(left panel)** and measured by three-dimensional echocardiography **(right panel)**. CO, cardiac output; EDV, left ventricular end-diastolic volume; ESV, left ventricular end-systolic volume; EF, ejection fraction; HR, heart rate; LV, left ventricular; MOD BP, biplane mode; SV, stroke volume; SpI, sphericity index.

From the apical four- and two-chamber views, the 2D LV volumes and EF were measured offline by a single experienced operator using the biplane method of disks’ summation (modified Simpson’s rule, Figure 1) (17). 3DE datasets of the LV were obtained from the apical approach using multi-beat full-volume acquisition during breath-holding and taking care to encompass the entire LV cavity in the dataset (16, 21). 3D LV volumes and EF (Figure 1) were measured offline by a single experienced operator using a dedicated software package for the LV analysis (4D AutoLVQ, GE Healthcare, Horten, NO). Measurement workflow started with the semi-automated detection of the LV endocardial borders. When needed, manual editing was used to optimize the endocardial contour identification (22). To trace the endocardial borders of both the 2DE and 3DE datasets, the end-diastolic frame was selected as the frame before the mitral valve closure, whereas the end-systolic frame was identified as the frame before mitral valve opening.

### Follow-up and study endpoints

The primary clinical endpoint was the occurrence of death for any cause. The secondary endpoint was the composite of all-cause death and hospitalization for cardiac indication (either from heart failure, acute coronary syndromes, or arrhythmias). Information concerning both survival and hospitalization were obtained at regular intervals via: (*i*). review of electronic medical records of regular outpatient visits and hospital admission records; (*ii*). telephone interview with the patient, or if deceased, with family members; and (*iii*). contact with the patient’s physicians. Mortality status was verified independently through the Social Security Death Index and death certificates. For patients without events, the date of the last contact was used for survival analysis. Assignment of clinical events was performed by physicians unaware of the patients’ echocardiographic and clinical characteristics.

### Statistical analysis

The normal distribution of continuous variables was tested with Kolmogorov–Smirnov test. Continuous variables were expressed as mean ± SD or as median (interquartile range). Categorical variables were expressed as absolute numbers (percentages). We compared the clinical and echocardiographic characteristics between patients who died and those who survived. Student’s t test for independent samples was used to compare differences between two groups of normally distributed continuous variables, while Mann-Whitney’s test was used to compare differences between two groups of non-normally distributed continuous variables. The chi-square test was used to assess differences between two groups of categorical variables. Pearson correlation coefficient was used to assess correlations between 2DE and 3DE parameters. Kaplan Meier curves were constructed to assess the prognostic stratification for the different 2DE and 3DE parameters. The log-rank test was used to assess the statistical significance between strata.

Since there was a high correlation between 2DE and 3DE LV volumes and EF, we built several pairs of models by separately adding the 2DE and 3DE parameters into the baseline clinical model. The first step used only the clinical variables available in our cohort (thus excluding the echocardiographic parameters), in a stepwise Cox proportional-hazard model, censoring data at first event. The clinical variables with significance level < 0.05 were included into the multivariate baseline clinical model (CM). Next, the 2DE parameters were added sequentially to the baseline CM, and then we built a third model by adding sequentially the 3DE parameters to the baseline CM, resulting in pairs of models for the end-diastolic volume (EDV), the end-systolic volume (ESV), and EF. The proportional hazards assumption for the Cox regression models was verified by visual assessment of Kaplan-Meier curves. The whole process was repeated for both the all-cause death and the composite endpoint. The independent and incremental value of each model compared to the previous one was assessed by comparing model χ^2^ statistics. Model discrimination was further assessed using Harrel’s C-index. Comparison of the C statistics of the various multivariable models was performed using the method proposed by Newson RB (23).

Time-dependent Receiver Operating Characteristics (ROC) curve analyses for censored event times were used to compare the prognostic value of the LVEF severity grading threshold values obtained with 2DE and 3DE and Areas Under Curve (AUC) were derived. The De Long test was used to compare the AUCs of tested threshold values (24).

Data were analyzed using SPSS v.24.0 (SPSS Inc., Chicago, IL, USA) and MedCalc 20, (MedCalc Software Ltd., Ostend, Belgium). Statistical significance was defined as *p* < 0.5.

## Results

### Clinical data and outcome

**FIGURE 2:**
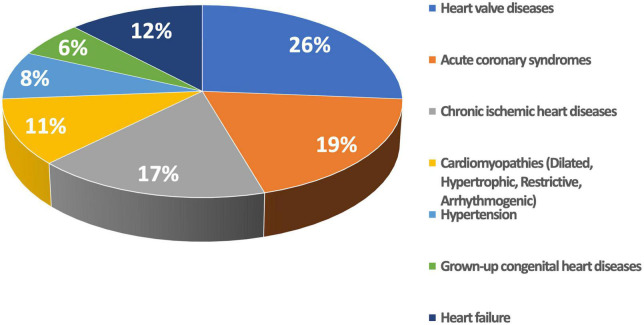
Frequency of the different clinical indications for the echocardiographic study.

The final study population included 725 patients with various cardiac conditions (Table 1) who underwent echocardiography for various clinical reasons (Figure 2).

During the follow-up period of (median 3.39 years, IQR = 2.6 years), 111 (15.3%) deaths (83 of them, 75% were cardiac deaths), and 248 (34.2%) cardiac hospitalizations occurred. The latter were distributed as follows: 151 (20.8%) for heart failure, 51 (7.0%) for acute coronary syndrome, and 46 (6.3%) for arrhythmias. Finally, 304 (41.9%) patients reached the composite endpoint of all-cause death or cardiac hospitalization.

Patients who died were older and had higher heart rate than survivors (Table 1). In addition, patients who died had a higher prevalence of diabetes, chronic kidney disease, anemia, permanent atrial fibrillation, and history of ischemic heart disease than the survivors (Table 1). Conversely, sex and body mass index distribution, as well as the prevalence of smoking, hypertension, and dyslipidemia were similar between the two groups.

### Comparison between 2D and 3D echocardiography and association with outcomes

As expected, there was a close correlation between the 2DE and 3DE LV EDVs (*r* = 0.964; *p* < 0.001), ESVs (*r* = 0.972; *p* < 0.001), and EFs (*r* = 0.925; *p* < 0.001). However, 3DE LV volumes were larger than the 2DE LV volumes (bias = + 21 ml, LOI ± 18 ml for the EDV, and bias = + 19 ml, LOI ± 10 ml for the ESV, respectively), whereas the 3D LVEF was lower than the 2D LVEF (bias = −2%, LOI ± 10).

2DE and 3DE LV volumes were categorized into normal or dilated according to technique-specific threshold values. The incidence of LV dilatation was significantly higher when LV EDV and ESV were calculated by 2DE than when they were measured with 3DE (45% vs. 35%, *X*^2^ = 12.93; *p* = 0.0003 and 56 vs. 51%, *X*^2^ = 4.237; *p* = 0.034, respectively).

**FIGURE 3:**
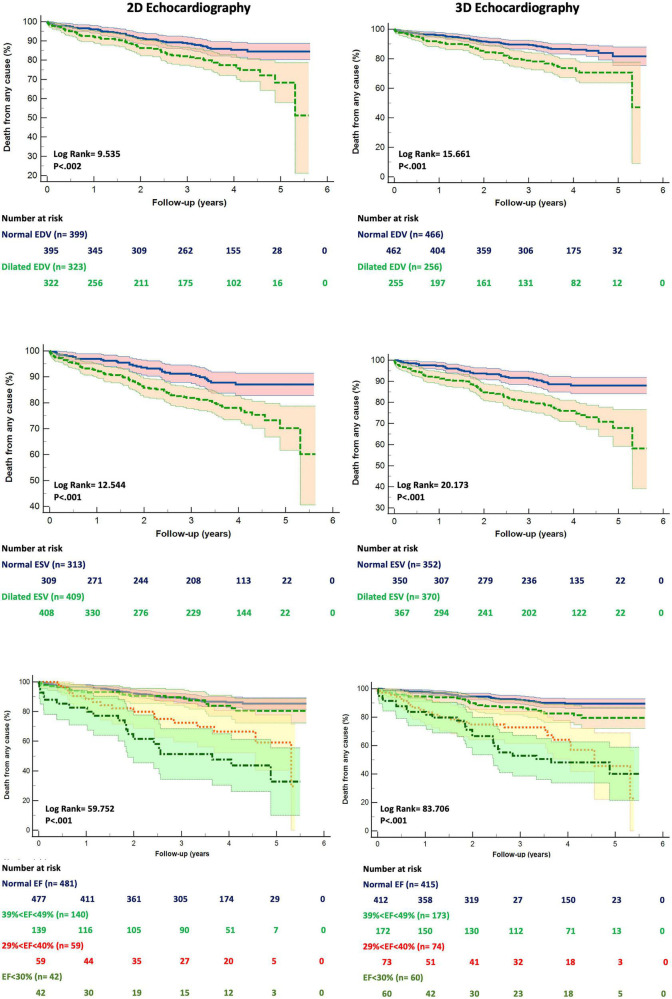
Kaplan Meyers survival curves for dilated left ventricular end-diastolic **(upper panels)**, end-systolic **(mid panels)** volumes and reduced ejection fraction **(bottom panels)** obtained by two- **(left panels)** and three-dimensional **(right panels)** echocardiography, respectively. Abbreviations as in Figure 1.

**FIGURE 4:**
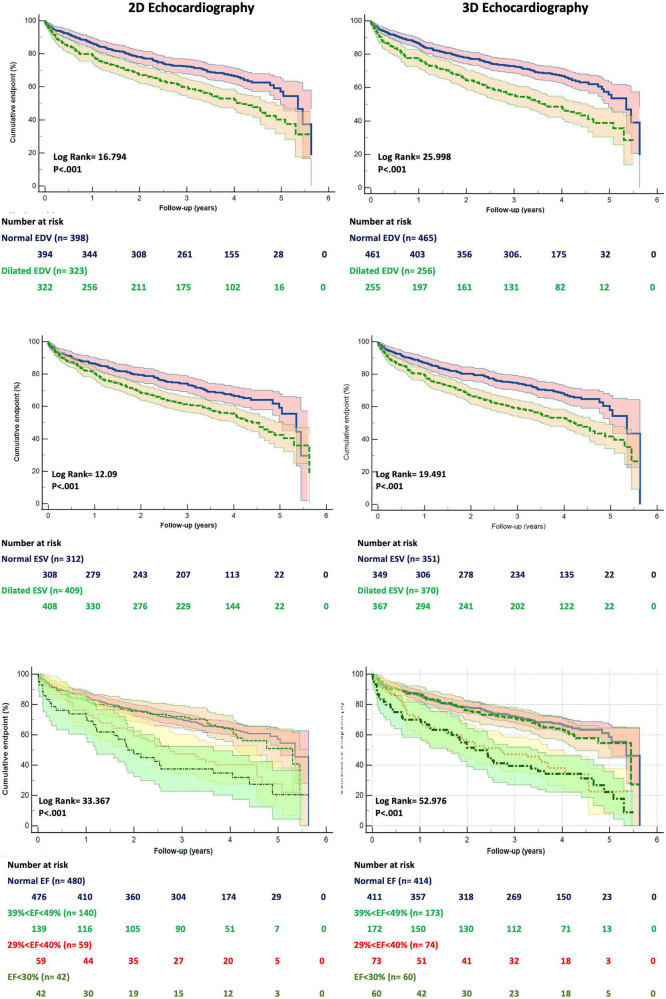
Kaplan Meyers curves for freedom from the cumulative event of death and cardiac hospitalization for dilated left ventricular end-diastolic **(upper panels)**, end-systolic **(mid panels)** volumes and reduced ejection fraction **(bottom panels)** obtained by two- **(left panels)** and three-dimensional **(right panels)** echocardiography, respectively. Abbreviations as in Figure 1.

The patients who died had larger LV EDV and ESV, as well as lower EF, by both 2DE and 3DE than the survivors (Table 1). Although 2DE LV volumes were associated to worse all-cause death survival and event-free survival, 3DE LV volumes were stronger predictors of both. 3DE EDV ≤ 85 ml/m^2^ in men and ≤ 78 ml/m^2^ in women (*X*^2^ = 15.661; *p* < 0.001) and 3DE ESV ≤ 34 ml/m^2^ in men and ≤ 28 ml/m^2^ in women (*X*^2^ = 20.173; *p* < 0.001) were more strongly associated (all *p* = 0.001) with all-cause death than either 2DE EDV ≤ 74 ml/m^2^ in men and ≤ 61 ml/m^2^ in women (*X*^2^ = 9.595; *p* = 0.002) or 2DE ESV ≤ 31 ml/m^2^ in men and ≤ 24 ml/m^2^ in women (*X*^2^ = 12.544; *p* < 0.001) (Figure 3). Similarly, 3DE EDV ≤ 85 ml/m^2^ in men and ≤ 78 ml/m^2^ in women (*X*^2^ = 25.998; *p* < 0.001) and 3DE ESV ≤ 34 ml/m^2^ in men and ≤ 28 ml/m^2^ in women (*X*^2^ = 19.491; *p* < 0.001) were more strongly associated (all *p* = 0.001) with the composite endpoint of all-cause death or cardiac hospitalizations than either 2DE EDV ≤ 74 ml/m^2^ in men and ≤ 61 ml/m^2^ in women (*X*^2^ = 16.794; *p* = 0.002) or 2DE ESV ≤ 31 ml/m^2^ in men and ≤ 24 ml/m^2^ in women (*X*^2^ = 12.092; *p* < 0.001) (Figure 4).

### Survival analysis based on LVEF ranges derived from 2DE and 3DE measurements

We divided our study population into four groups, according to the ranges of the LVEF recommended by current guidelines: normal LVEF (> 52% for men, > 54% for women), mildly reduced LVEF (51-41% for men, 53%-41% for women), moderately reduced LVEF (40-30% for men and women), and severely reduced LVEF (< 30% for men and women). The grading of LV EF was more severe when 3DE was used to measure LV volumes (*X*^2^ = 13.22; p = 0.0042, Figure 3) than when LV volumes were calculated using 2DE.

Kaplan Meier survival curves were derived for the different ranges of the 2DE LVEF and 3DE LVEF, respectively (Figures 3, 4).

Both 2DE and 3DE LVEF curves could significantly stratify the risk of death among the different ranges of the LVEF. However, the Kaplan Meier curves for the 3DE LVEF thresholds of LV dysfunction had a higher χ^2^ at the log-rank discrimination analysis by comparison with the 2DE LVEF threshold values (χ^2^ = 83.706, p < 0.001 vs. χ^2^ = 59.752, p < 0.001). Moreover, the Receiver Operating Curves for both 2DE and 3DE LVEF threshold values showed a higher area under the curve (AUC) for the 3DE LVEF ranges than for the 2DE LVEF ranges in predicting death (0.76 ± 0.03 vs. 0.69 ± 0.04; *p* < 0.001).

Furthermore, only the 3DE LVEF was a significant predictor of time-to-event for each change in the LVEF category [2DE LVEF: HR 1.33 (0.88-2.01), *p* = 0.169; 3DE LVEF: HR 0.35 (0.24-0.52), *p* < 0.001]. Moreover, the HR increase for the three categories with reduced LVEF compared with the group with normal LVEF were higher when using the 3DE LVEF threshold values (mildly reduced LVEF: HR 2.40 (1.23-4.65), *p* = 0.01; moderately reduced LVEF: HR 7.68 (4.15-14.24), *p* < 0.001; severely reduced LVEF: HR 10.4 (5.67-19.09), *p* < 0.001; χ^2^ = 98.26, *p* < 0.001) than when using the 2DE LVEF ones (mildly reduced LVEF: HR 1.46 (0.77-2.77), *p* < 0.25; moderately reduced LVEF: HR 3.68 (2.03-6.7), *p* < 0.001; severely reduced LVEF: HR 7.66 (4.43-13.25), *p* < 0.001; χ^2^ = 77.10, *p* < 0.001).

### Incremental value of left ventricular volumes and ejection fraction to predict outcome

The clinical parameters associated with all-cause death and the composite endpoint are listed in Table 2. Clinical parameters associated with all-cause death were age, dyslipidemia, permanent atrial fibrillation, ischemic heart disease, and serum levels of creatinine and hemoglobin (Table 2). Clinical parameters associated with the composite endpoint of all-cause death and cardiac hospitalization were permanent atrial fibrillation, ischemic heart disease, and serum levels of creatinine and hemoglobin (Table 2).

**TABLE 2:** Demographic and clinical parameters associated with both all-cause death and the composite endpoint of death and cardiac hospitalization.

	All-cause death HR (95% CI)				Composite endpoint HR (95% CI)			
	**Univariate**	***P***	**Multivariate** **(*X*^2^ = 242.56)**	***P***	**Univariate**	***P***	**Multivariate** **(*X*^2^ = 131.57)**	***P***
Age	1.031 (1.015-1.047)	**<0.001**	1.031 (1.015-1.047)	**< 0.001**	1.002 (0.993-1.01)	0.677	−	**−**
Gender	1.040 (0.68-1.59)	0.856	−	−	0.929 (0.718-1.204)	0.979	−	−
Body mass index	0.985 (0.931-1.043)	0.615	−	−	0.989 (0.753-1.286	0.907	−	−
Diabetes	1.029 (0.439-1.041)	0.905	−	−	0.915 (0.668-1.254)	0.581	−	−
Hypertension	0.676 (0.390-0.886)	0.076	−	−	0.984 (0.753-1.286)	0.907	−	−
Dyslipidemia	0.588 (0.387-1.013)	**0.011**	0.581 (0.387-0.873)	**0.009**	0.869 (0.676-1.118)	0.274	−	−
Atrial fibrillation	1.856 (1.202-2.867)	**0.005**	1.874 (1.215-2.889)	**0.004**	2.003 (1.529-2.623)	**< 0.001**	2.014 (1.552-2.614)	**<0.001**
Ischemic heart disease	2.253 (1.311-3.872)	**0.003**	2.247 (1.331-3.793)	**0.002**	1.776 (1.254-2.516)	**0.001**	1.659 (1.195-2.304)	**0.003**
Creatinine	1.003 (1.001-1.005)	**<0.001**	1.003 (1.001-1.005)	**<0.001**	1.003 (1.001-1.004)	**< 0.001**	1.003 (1.001-1.004)	**<0.001**
Hemoglobin	0.969 (0.958-0.980)	**<0.001**	0.968 (0.955-0.979)	**<0.001**	0.980 (0.983-0.997)	**0.004**	0.989 (0.982-0.995)	**<0.001**

CI, confidence interval; HR, hazard ratio. Bold values represent the statistically significant.

**FIGURE 5:**
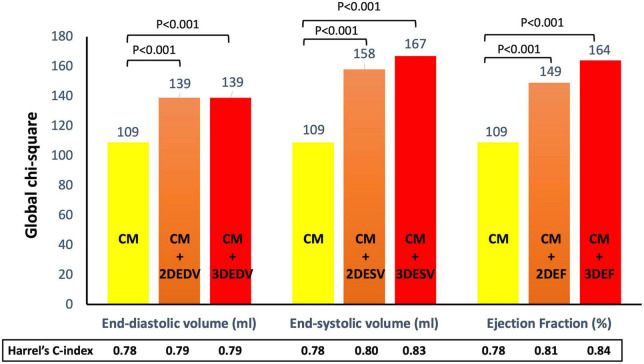
Addition of left ventricular volumes and ejection fraction obtained from both two- and three-dimensional echocardiography significantly increased the association with all-cause death of the clinical model based on permanent atrial fibrillation, ischemic heart disease, and serum levels of creatinine and hemoglobin. Both three-dimensional end-systolic volume and ejection fraction had stronger association with outcome than the corresponding two-dimensional parameters. CM, clinical model; EDV, end-diastolic volume; EF, ejection fraction; ESV, end-systolic volume.

Clinical predictors identified at multivariable analysis as independently associated with outcome were placed in two separate stepwise regression models (CM_death_ and CM_composite_) for all-cause death and the composite endpoint, respectively. For all-cause death, the Harrel’s C-index (HC) of the CM_death_ was 0.78 (95%CI 0.72-0.84). The addition of the EDV to CM_death_ increased the HC at the same value using both 2DE (HC = 0.79, 95%CI 0.73-0.85) and 3DE (HC = 0.79, 95%CI 0.73-0.85). Conversely, the addition of ESV (HC = 0.83, 95%CI 0.74-0.91) and EF (HC = 0.84, 95%CI 0.78-0.9) by 3DE was associated with greater HC than the same parameters obtained with 2DE (HC = 0.80, 95%CI 0.75-0.85, and HC = 0.81, 95%CI 0.76-0.86) (Figure 5).

**FIGURE 6:**
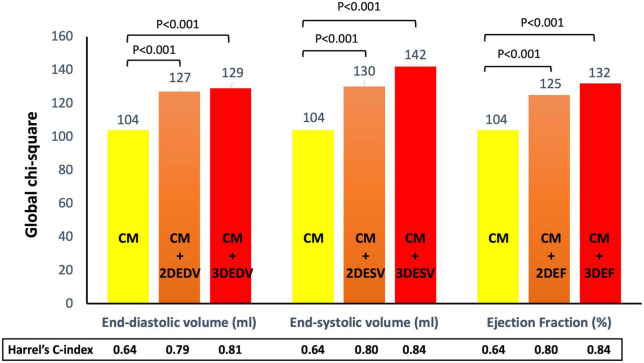
Addition of left ventricular volumes and ejection fraction obtained from both two- and three-dimensional echocardiography significantly increased the association with the composite endpoint of all-cause death and cardiac hospitalization of the clinical model based on age, dyslipidemia, permanent atrial fibrillation, ischemic heart disease, and serum levels of creatinine and hemoglobin. Both three-dimensional end-systolic volume and ejection fraction had stronger association with outcome than the corresponding two-dimensional parameters.

For the composite endpoint, the HC of the CM_composite_ was 0.64 (95%IC 0.61-0.68). The addition of the EDV by 2DE increased the HC of the model (0.79, 95%CI 0.73-0.85), but the 3DE EDV was associated with a larger increase of the HC (0.81, 95%CI 0.75-0.88). Conversely the increase of the HC of the CM_composite_ obtained by adding the ESV and EF was similar for the 2DE (HC = 0.80, 95%CI 0.74-0.85) and HC = 0.84, 95%CI 0.77-0.92, respectively for 2DE ESV and EF) and 3DE (HC = 0.80, 95%CI 0.75-0.86) and HC = 0.84, 95%CI 0.78-0.90, respectively for 3DE ESV and EF) (Figure 6).

## Discussion

Our study shows that, in unselected patients undergoing clinically indicated routine echocardiography, the LV volumes and EF measured with 3DE were more strongly associated with outcome than the same parameters calculated by 2DE. The main findings of our study can be summarized as it follows: (1) As expected, in addition to the clinical predictors (age, anemia, chronic kidney disease, atrial fibrillation, and ischemic heart disease), both LV volumes and EF were associated with both all-cause mortality and the composite endpoint of death and cardiac hospitalization; (2) Survival analysis based on LV dilation according to 2DE and 3DE measurements showed that the 3DE threshold values for LV dilation had a higher discriminative power than the 2DE cutoff values in predicting both all-cause death and the cumulative endpoint of death and cardiac hospitalization; (3) Survival analysis based on LVEF threshold values for defining LV dysfunction severity showed that the LVEFs measured by 3DE have higher discriminative power for all-cause mortality and the composite endpoint of death and cardiac hospitalization than the 2DE ones; (4) When added to the baseline clinical model developed for all-cause death (i.e., CM_death_) both 3DE LVESV and EF were more strongly associated to the occurrence of all-cause mortality than the corresponding 2DE parameters.

### Prognostic value of clinical and echocardiographic data

According to the reports of epidemiologic studies, cardiovascular diseases are the leading cause of death globally, and, particularly, in the most developed countries (25). In the European Union, cardiovascular diseases cause 35% of the deaths in women and men under the age of 75 years (26). In the next decade, the expected number of disability-adjusted-life-years that will be lost because of cardiovascular diseases will increase from 169 million in 2020 to 187 million in 2030^1^.

Cardiovascular morbidity and mortality in the general population have been related to both non-modifiable (e.g., age, sex, genetics) (27) and potentially modifiable risk factors (28), and to the underlying cardiac condition^2^. Accordingly, in our study patients, age, chronic kidney disease, anemia, atrial fibrillation, and ischemic heart disease were significantly associated with clinical outcomes. In addition, also the LV volumes and EF measured by both 2DE and 3DE have been associated with clinical outcomes (19, 29, 30).

Non-invasive assessment of LV volumes and EF are critically important for clinical decision-making and represents the most frequent indication for an echocardiographic study. Eligibility to device implantation of patients with LV dysfunction, discontinuation of potentially cardiotoxic chemotherapy in cancer patients, indications to cardiac surgery or to treatment initiation in asymptomatic patients are among the most important clinical decisions that rely on an accurate measurement of LV EF. LV volume calculations by 2DE is highly operator dependent, uses only limited data contained in a few predetermined tomographic planes of the LV to assess global myocardial function, and relies on geometrical assumptions that may not be necessarily valid in every patient. The geometric assumptions about LV shape associated with the 2DE algorithms make the calculations of LV volumes and EF more inaccurate in patients in whom this information is more critical (i.e., patients in whom the LV geometry is distorted because of aneurysms, or in those with extensive wall motion abnormalities) (31, 32). With 3DE, LV volumes are actually measured (and not calculated anymore) without any assumption regarding LV shape (14). This technique has been extensively validated using the cardiac magnetic resonance as a reference modality (10, 15), and was demonstrated to be more timesaving, reproducible, repeatable and accurate than conventional 2DE for both LV volumes and EF measurements (11, 33–35).

Caselli et al. (36) showed that, in a limited cohort of 178 patients, 3DE LV volumes and EF had a significant association with the composite endpoint of death, myocardial infarction or stroke but not with the primary endpoint of cardiovascular death. Similarly, Mancuso et al. (37) showed that, in 89 patients with systolic heart failure, the LVEF measured by 3DE was an independent predictor for a composite endpoint of death, cardiac transplantation and hospitalization for HF, whereas LV volumes were not. However, in addition to the smaller number of patients and the specific clinical settings of this study, it should be noted that, in contrast with available data, the LV volumes measured by 3DE were smaller than those obtained with 2DE. In our study, the LV volumes measured by 3DE were significantly larger than those obtained by 2DE, in accordance to other studies that showed the higher accuracy of the 3DE for the measurement of the LV volumes in comparison to cardiac magnetic resonance (10). New 3DE technologies combined with an increased experience in 3DE of the cardiologists who perform echocardiography allow encompassing larger LVs into the 3DE dataset at a good volume rate, allowing good endocardial delineation and better measurements of the LV volumes. A previous study performed in unselected patients with a wide range of LV volumes showed that the 3DE LV volumes measured by different echocardiography systems were similar and had a better accuracy than 2DE when compared to cardiac magnetic resonance (38).

The present study adds to previous ones by showing that LV volumes and EF measured by 3DE provide incremental prognostic value over 2DE also in patients referred for a routine and clinically-indicated echocardiography study. Both LV volumes and EF calculated by 2DE were independently associated with either cardiac death or the composite of cardiac death and hospitalization for cardiac causes after adjusting for covariates. However, 3DE LV ESV and EF were able to significantly increase the power of the predictive model when added to a model including the clinical variables. These findings suggested that LV ESV and EF obtained from 3DE were superior to those derived from 2DE to predict cardiac death and the need of hospitalization for cardiac issues.

### Survival analysis based on LVEF threshold values derived by 2DE and 3DE

When using LVEF values obtained from 3DE to grade the extent of LV dysfunction severity into normal function, and mild, moderate and severe LV dysfunction, LVEF measured by 3DE showed higher discriminant power for survival than LVEF measured by 2DE. These results are consistent with the findings by Stanton et al. (19) who compared the occurrence of the composite endpoint between patients with “normal” and “abnormal” LVEF measured by 2DE and 3DE. LVEF measured by 3DE was also an independent predictor of major arrhythmic events and improved the ability to predict the arrhythmic risk in 172 patients with LVEF below 50% (39). In that study, when compared with 2DE LVEF, 3DE-measured LVEF changed the indication to implantable cardioverter-defibrillator implant in 20% of the patients. Finally, in our patients, only the 3DE measurement of the LVEF was an independent predictor of time to event for each change in the LVEF.

### Survival analysis based on LV dilation according to 2DE and 3DE

The 3D LVEDV threshold values used to identify patients with dilated LV provided better stratification of the risk of death compared to 2D LVEDV. Dilated LVESV according to the 3DE cut-offs proposed by Muraru et al. (21) in normal individuals showed to be a predictor for time to event, as well, while the 2D LVESV cut-offs offered by the current guidelines for LV dilation did not (17).

### Clinical implications

Our findings showed that, although LV volumes obtained by 2DE and 3DE are significantly correlated, 3DE might be better than 2DE in assessing the severity of LV dysfunction and the prognosis of the patients referred to the echocardiography laboratory for routine, clinically indicated study, and physicians should use parameters measured by 3DE to better guide patients’ management. Future research should focus on whether 3DE can improve the predictive value in larger and prospective cohorts of consecutive patients, and whether 3DE guided therapy can improve clinical outcomes.

## Limitations

This study has several limitations. This study was single-center and retrospective, and no causal relationship can be established from our findings. Second, only patients with stable clinical conditions and good quality 2DE and 3DE datasets were enrolled in the study, and whether these findings can be extrapolated to the general population of consecutive patients that are examined in the echocardiography laboratory remain to be established. Finally, this study was carried on in tertiary center with a long-standing experience in transthoracic 3DE. Whether our results can be applied to the generality of the echocardiography laboratories require further prospective and multicenter studies.

## Conclusion

In patients referred to the echocardiography laboratory for a clinically indicated, routine echocardiography study, 3DE was a better predictor than 2DE of both cardiac death and the composite of cardiac death and hospitalization for cardiac cause. Our findings support the recommendation made by the European Association of Cardiovascular imaging and American Society of Echocardiography that, in laboratories with experience and equipment, 3DE should be used for LV volume and EF measurements and implemented into the clinical routine of the echocardiography laboratory.

## Data availability statement

The raw data supporting the conclusions of this article will be made available by the authors, without undue reservation.

## Ethics statement

The studies involving human participants were reviewed and approved by Ethics Committee of the Istituto Auxologico Italiano, IRCCS. Written informed consent for participation was not required for this study in accordance with the national legislation and the institutional requirements.

## Author contributions

DM and LB developed the concept, designed the study, drafted the first manuscript, and approved its final version. SB and DG prepared the database, made all the statistical analyses, and revised the manuscript critically. NR, MG, FP, MT, EC, and GP critically revised the manuscript draft for important intellectual content. CS and CB prepared the figures, drafted the tables, and revised the manuscript draft for important intellectual content. All authors have approved the final manuscript that has been submitted.

## Footnotes

1
https://apps.who.int/iris/handle/10665/43007

2
https://ec.europa.eu/eurostat/statistics-explained/index.php?title=Cardiovascular_diseases_statistics
